# So Far So Good: Emotion in the Peripersonal/Extrapersonal Space

**DOI:** 10.1371/journal.pone.0049162

**Published:** 2012-11-21

**Authors:** Berenice Valdés-Conroy, Francisco J. Román, Jose A. Hinojosa, S. Paul Shorkey

**Affiliations:** 1 Departamento de Psicología Básica 1, Universidad Complutense de Madrid, Madrid, Spain; 2 Departamento de Psicología Biológica y de la Salud, Universidad Autónoma de Madrid, Madrid, Spain; 3 Nuffield Department of Clinical Neurosciences, University of Oxford, Oxford, United Kingdom; University of Bologna, Italy

## Abstract

Current accounts of spatial cognition and human-object interaction suggest that the representation of peripersonal space depends on an action-specific system that remaps its representation according to action requirements. Here we demonstrate that this mechanism is sensitive to knowledge about properties of objects. In two experiments we explored the interaction between physical distance and object attributes (functionality, desirability, graspability, etc.) through a reaching estimation task in which participants indicated if objects were near enough to be reached. Using both a real and a cutting-edge digital scenario, we demonstrate that perceived reaching distance is influenced by ease of grasp and the affective valence of an object. Objects with a positive affective valence tend to be perceived reachable at locations at which neutral or negative objects are perceived as non-reachable. In addition to this, reaction time to distant (non-reachable) positive objects suggests a bias to perceive positive objects as closer than negative and neutral objects (exp. 2). These results highlight the importance of the affective valence of objects in the action-specific mapping of the peripersonal/extrapersonal space system.

## Introduction

One of the major goals of psychology is the understanding of human-object interactions. In our daily lives we manipulate and interact efficiently with a multitude of objects around us. Imagine you are at a dinner table with some friends and you are asked to hand the salt to someone near you. Your brain estimates the distance, size, shape, and weight of the object and combines this information with postural information from your body to organize the movements to reach that object. Furthermore, the person who asked knew that the object was within your reaching distance and that it may be easy for you to grasp. How does the brain combine visuospatial and proprioceptive information in order to execute our own actions and predict the actions of others? Do we have a specialized unitary system to store and organize body-object interactions in physical space? If so, what are the neurological, environmental and psychological variables that modulate this system?

During the last thirty years, modern neuroscience has provided important evidence indicating that the brain has a specialized system to represent the so-called *near-space* or *peripersonal space.* Single cell studies with non-human primates have described a type of cell with multimodal receptive fields located in the ventral intraparietal cortex, the putamen and in a small region of the premotor area of the monkeýs brain. These cells respond to tactile stimulation of the hand or face and also to visual and auditory stimuli that co-occur within 20 to 30 centimeters of the tactile receptive field [1]–[3], (for a review see [4]). The body-centered activity of these cells constitutes the representation of peripersonal space. Recent behavioural and neuroimaging studies with healthy volunteers suggest that this multimodal system also exists in the human brain [5]–[10] (see Macaluso & Maravita [11] for a review). Studies with brain-damaged patients have described some cases of patients who are specifically impaired in detecting information within the peripersonal space [12]–[13], or show tactile extinction when a competing stimulus of a different modality is presented [14].

The multimodality of the peripersonal space system has been proposed to be related to the control of movements in the space [3] and, in particular, with a general attentional function of the system dedicated to monitoring the approach of objects towards the body [1]. Most of the research in this area has focused on studying the extent to which the properties of this neuronal system are fixed or not, and it has been demonstrated that this peripersonal space mapping can be modified with learning and through the use of tools as extensions (see [15]–[17]). Using a line bisection task, Berti and Frassineti [18] described the case of a patient with near-space neglect whose neglect symptoms were transferred into the far-space after the use of an extension tool, as if the use of this tool remapped the far space as near/reachable, peripersonal space. Similarly, in the linguistic domain, Coventry, Valdés, Castillo & Guijarro-Fuentes [19] have demonstrated that the use of spatial demonstratives (e.g., this, that) when referring to objects in the space depends not only on the real physical distance but also on other parameters such as previous interaction with the object. As did Berti and Frassineti [18], Coventry et al. [19] also found that when using an extension tool participants increased their use of the word *this* to refer to objects that fell into “extrapersonal space”.

Both behavioral and neuroimaging research with healthy volunteers have accumulated a good deal of evidence about the interactions between perception and action. We know now that the solely observing or naming familiar tools activates not only visual but also premotor areas of the brain [20]–[21] suggesting that premotor cortex may have both motor and cognitive functions related to space perception, action understanding and imitation [22]. Visual perception of objects is also influenced by the knowledge about the functional properties of objects and their associated actions [23]–[24]. Specifically, it has been shown that presenting objects in difficult grasping positions increases perceived distance to those objects [25].

The *action-specific account* suggests that the perception of objects in a particular space is determined to a great extent by the actions we perform with them [26]. Stimulus-response compatibility effects have shown that reach-to-grasp actions are facilitated when a graspable object is presented in a compatible view as opposed to an incompatible view [27]. More recently, using a verb-picture verification task in a virtual reality scenario, Costantini, Ambrosini, Scorolli and Borghi [28] have shown that participants were faster to respond when objects (e.g. a bottle) were preceded by a functional verb (e.g. “to drink”) as compared to situations in which the verb referred only to observation (e.g. “to look at”). Importantly, this effect was obtained when the objects were presented in the reachable space. However, using a priming paradigm, McNair and Harris [29] found that naming accuracy was primed by grasping similarities but not by functional properties of objects, suggesting a strong automatic processing of motor action planning.

Similarly, the *postural stability account* [30] states that perceived reaching distance depends on our postural constrains. Ecological studies have shown that altering proprioceptive information (e.g., carrying a heavy backpack) causes an overestimation of egocentric distance (the distance from the object to the observer) [31]. Manipulating posture constraints has also been shown to have an important effect in the estimation of reaching limits [32]. As in distance estimation, perceiving what is reachable involves not only information about the objective location of objects but also information about our body and the possibilities to act on them.

In line with these findings, recent evidence comparing pointing and grasping actions suggests that the representation of the peripersonal space can be remapped according to specific action requirements during the process of the action [7].

Object-directed actions are linked to different consequences that determine to a great extent our knowledge of the world. It is surprising, then, that only a small number of studies have examined the influence of the affective properties of objects in the action-specific space system.

Some studies have demonstrated that the emotional valence of stimuli influences the speed and direction of movements [33]. Likewise, motor actions modulate subsequent affective evaluations of the objects involved [34]; see [35] for a review. In a recent study, Constable, Kritikos and Bayliss [36] demonstrated that other psychological concepts like ownership may also play important roles during object-oriented actions. Finally, egocentric distance estimation seems to be affected by the desirability of objects [37]. The results from this study can be interpreted in support of the *positivity-closeness hypothesis* [38], which posits a bias to perceive desirable objects as being located closer than non-desirable objects. Additionally, Ode, Winters and Robinson [39] have shown that the positive valence of words produces perceptual overestimations of size and exposure time of such words. Ode et al. [39] explained their results using the *incentive salience theory* which, similar to the *positivity-closeness hypothesis,* states that appealing locations of objects are perceived more vividly and may induce perceptions consistent with approach-motivated behaviors.

In the present study, we aimed to contribute to the investigation of the role of the affective properties of objects in reaching action programing. Our rationale was that the affective valence of objects should affect not only perceptual judgments about egocentric distance but also estimates during planning a particular action (in this case *reaching*).

We know that people tend to overestimate reaching; that is, people tend to think they can reach an object when in reality they are not able [32], [40]. We reason that this overestimation may be due to the fact that reaching estimation relies on a flexible specific-action system that takes into account not only objective egocentric distance but also other variables such as body posture, graspability aspects and knowledge of the functional and affective properties of objects.

Through two separate studies, we addressed the question of how different aspects of objects (i.e., functional and affective) might alter the internal mapping of the spatial perceptual system. Imagine that, in the dinner situation introduced above, instead of requesting the salt your friend points at a spider in the same position on the table. The proximity of this threatening object would trigger a specific organization of the movements needed to react rapidly and accurately. The question that follows then is, how exactly the properties of objects modulate the organization of actions and their representation in the peripersonal space.

We therefore aimed to examine how the emotional properties of objects modulate reachability estimates. In a pilot real scenario study (Experiment 1) we manipulated the functional and affective properties of objects. In Experiment 2, with the use of cutting-edge large display technology, we incorporated precision and reaction time (RT) measures while participants completed a reaching estimation task on a 52-inch (129 cm) tactile digital surface.

We begin from the assumption that the peripersonal system, a flexible action-specific mechanism specialized in the control of human-object interactions, will be particularly sensitive to the affective and functional properties of objects. Taking explicit reaching estimates measures as an indirect correlate of the near/far spatial mapping, we expected this to vary depending on the knowledge about functional and affective properties of objects.

## Experiment 1: Pilot Study

### Method

The aim of this study was to test the effect of the functional and affective properties of objects on reaching distance perception in a real scenario setup.

**Figure 1:**
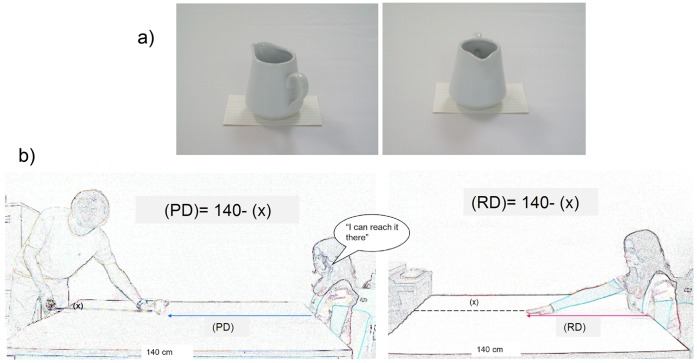
Real Scenario setup: a) example of object presentation (functional/no-functional orientation for a right-handed participant), b) procedure used to measure perceived distance (left) and reaching distance (right).

Forty undergraduate students (36 females) from the Universidad Complutense de Madrid (UCM) participated voluntarily in this study, giving written informed consent and obtaining course credit as compensation for their participation.The study had obtained previous approval by the Ethical Board of the Faculty of Psychology (UCM). All participants had normal or corrected-to-normal visual acuity. Participants sat at a square table (140×140 cm) that was covered with a plain white cover. Several objects were used to manipulate desirability (undesirable: used-like condom; desirable: 50€ note; neutral: white box), familiarity (familiar: participantś mobile phone; neutral: plain white box), orientation (tea jug in functional vs. non-functional orientations), and difficulty of reaching action (difficult: plain white box while holding a heavy book in one hand; easy: plain white box). Objects were placed at the edge of a white card (see Fig. 1a) and the experimenter slowly slid each object toward the participant. Sequence of object presentation was counterbalanced across participants. Participants had to indicate with a verbal response when the objects were close enough to be reached. Perceived reaching distances (PD) in centimeters were obtained for each object. Real reaching distances (RD) were measured by the experimenter at the beginning and at the end of experiment to control for discrete movements during the experiment. We then averaged the two measures to obtain a real reaching distance (see Fig. 1b). Misestimate ratios (MR) were also calculated from these two measures (PD/RD).

### Results

Table 1 shows the mean misestimate ratios obtained in each condition. An initial one-way Analysis of Variance (ANOVA) of misestimate ratios (MR) for all 7 objects showed a marginal trend, *F*(6, 234) = 2.054, *p* = .061, η^2^_p_ = .050. Participants overestimated their reaching distance to all objects (i.e., they reported the objects were within reach when in fact they were not). Post-hoc testing (LSD-Fisher) revealed that the tea-jug in a non-functional orientation was perceived significantly farther than the functionally-oriented, difficult, positive and negative objects (all *p<*.05.). Post-hoc comparisons of all other object dimensions (desirability, familiarity etc.) did not reach any significant effects.

**Table 1:** Mean misestimates ratios and Standard deviations (*SD*) obtained in Experiment 1.

	Functional	Non-Functional	Neutral	Neutral+weight	Familiar	Positive	Negative
*Mean*	1.22	1.2	1.22	1.22	1.21	1.23	1.22
*SD*	0.13	0.14	0.12	0.13	0.12	0.14	0.14

An independent sample of participants (*N* = 20; 14 female) evaluated the stimuli in terms of their valence, arousal and graspability. As mentioned above some studies suggest that motor planning for grasping may have an important effect in visual object recognition [29] and also in reaching distance estimates [25]. In addition, in this study we were primarily interested in the effects that emotion may play in reaching planning and peripersonal/extrapersonal space mapping. Using a Likert scale from 1 to 9, participants rated the most negative, low arousal and fine grasping objects as 1 and the most positive, high arousal and gross grasping objects as 9. Fine grasping and gross grasping were defined as grasping with the fingers (fine) or with the whole hand (gross). Table 2 shows the mean rating scores obtained for all objects employed in the study. ANOVA showed that objects could be grouped in statistically different categories in terms of their valence, *F*(4,80) = 34.675, *p*<.001; η^2^_p_ = .634, arousal *F*(4,80) = 24.513, *p*<.001; η^2^_p_ = .551 and graspability *F*(4,80) = 12.797, *p*<.001; η^2^_p_ = .390.

**Table 2:** Mean scores (*SD*) obtained for all objects employed in Experiment 1 for the three categories rated.

	Valence	Valence Group	Arousal	Arousal Group	Graspability	Grasp. Group
“used-like” condom	2.33 (2.01)	Negative	6.14 (2.15)	Medium	1.76 (2.15)	Fine
50€ note	7.71 (1.95)	Positive	7.57 (1.56)	High	2.38 (1.83)	Fine
Little white box	6.23 (1.44)	Neutral	4.33 (1.35)	Low	4.90 (1.84)	Gross
Tea-jug	6.09 (1.26)	Neutral	4.24 (1.44)	Low	4.67 (2.56)	Gross
Participant’s mobile phone	7.43 (1.48)	Positive	7.19 (1.33)	High	5.19 (2.27)	Gross

Post-hoc comparisons of *valence* ratings showed three identifiable groups: Negative (used-like condom, *M = *2.33*; SD = *2.01), Positive (Mobile phone and 50€ note, *M = *7.57*; SD = *1.20) and Neutral (Tea-jug and white box, *M = *6.16*; SD = *1.16). The same analysis for *arousal* ratings also yielded three identifiable groups: High arousal (Mobile phone and 50€ note, *M = *7.38*; SD = *1.21), Medium arousal (Used-like condom, *M = *6.14*; SD = *2.15) and Low arousal (tea-jug and white box, *M = *4.28*; SD = *.98). However, an analysis of the effects of these categories over estimate ratios showed no significant effects of *valence* or *arousal, F*(2,78) = 0.227; *p* = .781; η^2^_p_ = .006 Arousal and Valence produced the same groups of objects (Positive = high arousal objects, Negative = medium arousal object and Neutral = low arousal objects, see table 1).

*Graspability* ratings revealed two statistically different groups, *F*(4,80) = 12.797, *p*<.001; η^2^_p_ = .390, where fine grasping objects were the used-like condom and the 50€ note, (*M = *2.33*; SD = *2.01) and gross grasping objects were the mobile phone, the white box and the tea-jug, (*M = *7.57*; SD = *1.20). Analysis of the effect of this category over estimate ratios showed no significant effects *t*(39) = 1.380; *p* = .175; *d* = 0.078.

## Experiment 2: Digital Scenario

Experiment 1 showed some evidence of the effect of object properties in reaching distance estimates (concretely the ease of grasp of a functional object). There were a few obvious issues of note, however, that could have influenced the results. First, it was the experimenter who was always manipulating the objects. Second, as shown recently [36], property also interacts with our manipulations with objects in the space affecting the proximity of our actions. Finally, the lack of control in the speed at which the experimenter moved the objects towards the participants as well as variable RT to participants’ instruction to stop may have also influenced the reaching estimates. These were important aspects that, once identified in Experiment 1, motivated the performance of Experiment 2 within a PC-controlled digital environment in which the selection of stimuli based on affective valence and arousal was carefully controlled (Experiment 2a) and experimenter speed effects were not present (Experiment 2b).

### Experiment 2a: Materials Selection

**Figure 2:**
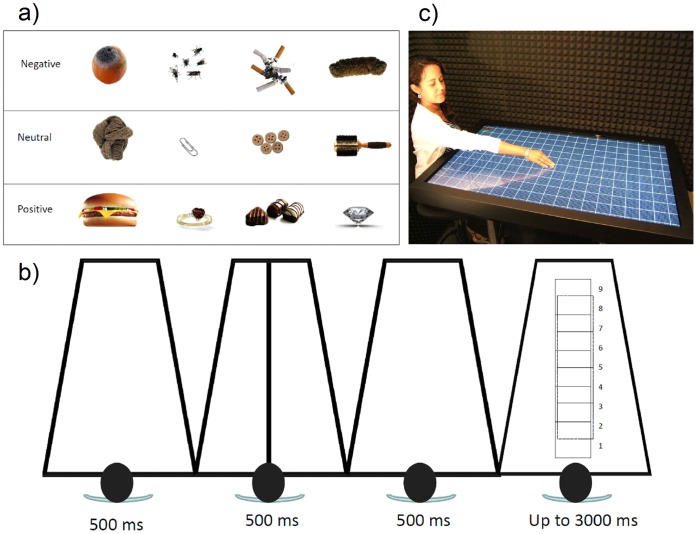
Digital scenario setup: a) example of stimulus positives, negatives and neutrals, b) example of trial and positions the 9 positions being 1 the nearest and 9 the furthest, c) example of reaching distances (RD) measured on the tactile surface. The Person shown here has given written informed consent (as outlined in the PLoS consent form) for publication of their photograph.

In a pre-test study, a group of 24 volunteer participants (19 female) that gave informed consent (also undergraduates from UCM) were asked to rate 30 objects. Using a Likert scale from 1 to 9, participants were asked to indicate how positive/negative and how activating/relaxing objects were, with 1 being very positive and 9 very negative for affective valence, and 1 very relaxing and 9 very activating for arousal. Each individual object was displayed in the center of a 17″ PC monitor in color over a white background. All stimuli were centered over a square of 328×294 pixels using a resolution of 1024×768 pixels. Below each object a scale from 1 to 9 was displayed as well as an indication of the type of judgment (e.g. *valence* or *arousal*). Presentation of objects was random and each object was presented once for evaluation of *arousal* and for evaluation of *valence*. Objects remained on the screen until participants typed their response. From the results of this study 12 objects were selected and grouped into three categories according to their level of affective valence: positive (*M* = 7.09, *SD = *0.67) negative (*M* = 1.94, *SD* = 0.29) and neutral (*M* = 5.19, *SD* = 0.52) Emotional objects were selected so that both positive and negative categories had the same level of arousal (*M* = 6.16, *SD* = 0.17; *M* = 6.61, *SD* = 0.27 positive and negative respectively) which was significantly different from neutral objects (*M* = 4.36, *SD* = 0.20), *F*(2,6) = 110.52, *p*<.01, η^2^_ p_ = .97. Bonferroni comparison showed that neutral objects were significantly different to negative and positive objects (both *p*<.05). All objects were significantly different in terms of their affective valence, *F*(2,6) = 85.38, *p*<.001, η^2^_ p_ = .96, (see Figure 2a).

Taking into account the possibility that grasping properties of objects may also have an effect on reaching estimates, we asked an independent sample of participants (*N* = 20; 14 female) to rate the objects in terms of their graspability. As in Experiment 1 participants used a Likert scale from 1 to 9 (where 1 = fine graspability and 9 = gross graspability).

Graspability ratings showed three groups that were statistically different *F*(2,40) = 40.416, *p*<.001, η^2^_ p_ = .669. Bonferroni comparisons showed significant differences between the three levels of graspability, *p*<.05. The fine grasping objects category consisted of chocolates, a paper-clip, cigarette-buds, buttons, flies and a ring (*M* = 2.63, *SD* = 1.01). Medium grasping objects were the excrement and the large diamond (*M* = 3.54, *SD* = 1.47). Finally, gross grasping objects were the knitting ball, hamburger, brush, and rotten orange (*M* = 5.96, *SD* = 1.53).

### Experiment 2b: Digital Scenario

23 undergraduate students (15 females) participated voluntarily in the study, giving written informed consent and obtaining course credit as compensation for their participation. Using a 52-inch digital tactile PC (1920×1080p; 125 cm long), the 12 selected objects from pretest (four positive, four negative and four neutral; see Fig. 2a) were presented in nine different square positions (200×200p; 13×13 cm) along the screen. Participants sat at the edge of the PC, which was positioned horizontally as if they were sitting at a table. Keeping hands under the screen, participants had to indicate with a wireless mouse if objects were close enough to be reached. It is important to mention that participants grabbed the mouse not as we normally do but with both hands as if it was a game button-press device. Previous studies have shown that the use of a mouse can extend the peripersonal space around the hand used to operate it [41]. Participants used their thumbs to respond by pressing the left or right button with their left or right thumb and keeping their hands below the tactile PC at all times. “Yes” and “No” response bottoms were counterbalanced across participants.

Each object appeared randomly at each position 5 times, resulting in a total of 540 trials. Each trial started with a blank screen for 500 ms, after which a fixation black line appeared along the center of the screen for 500 ms. After another 500 ms of blank screen, the stimuli appeared and remained until either the participant responded or 3 seconds passed. At the end of the experiment, real reaching distances (RD) were measured on the tactile surface (see Fig. 2b and c). Accuracy was calculated by comparing perceived reaching distance with real reaching distance to the object.

### Results

**Figure 3:**
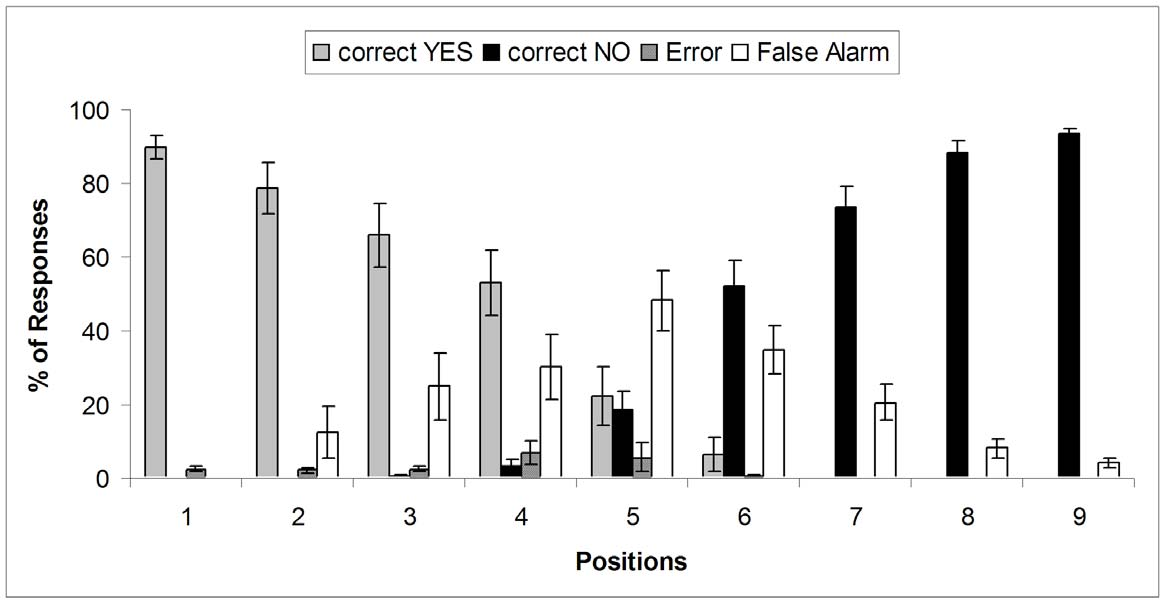
Accuracy data obtained in Experiment 2b. Locations 1 to 3 belong to region I (Near), 4 to 6 belong to region II (Middle) and 7 to 9 belong to region III (Far).

As expected, reaching estimation accuracy decreased in positions of more uncertainty. For a given trial a “Yes” response was considered accurate if the participants real reaching distance was greater than or equal to the current object position, with an error-range of 50 pixels. A “No” response to an event located beyond that “reach limit” was also considered accurate. As can be seen in Figure 3, reaching estimation accuracy was at chance level in positions 4, 5 and 6, where false alarms increased. Similar to Experiment 1, participants overestimated their reaching distance and pressed “Yes” at positions that were out of their reach. Following this accuracy pattern we divided the nine positions into three regions (I Near: 1 to 3, II Middle, 4 to 6 and III Far: 7 to 9).

Additional analyses were conducted in order to discard any individual item effects. Two-way ANOVA (9 Position × 12 Object) performed on RT and accuracy data showed no significant interactions.

RT analyses were computed only over accurate “Yes” responses in the near space region (region I: 78%), accurate “No” responses in the far space (region III: 81%) and accurate “yes and no” responses in middle space (region II: 52%). Please note that correct “No” responses in near space and correct “Yes” responses in far space were almost null (see Figure 3). A two-way ANOVA, 3×3 (region × valence) of mean RTs (<1500 ms and>250 ms) showed a significant interaction, *F*(4, 84) = 4.124, *p* = .004, η^2^_ p_ = .164 and a significant main effect of region *F*(2, 42) = 14.05, *p<*.001, η^2^_ p_ = .401. RTs at region I were significantly faster than in the other two regions. Note that degrees of freedom have changed cause *N* = 22. 1 participant didn’t have any correct responses in the middle region.

Individual one-way ANOVAs on each region revealed a significant effect of valence only at region III, *F*(2,44) = 7.381, *p* = .002, η^2^_ p_ = .251. Bonferroni post hoc testing revealed slower RT for positive objects in this region (see Table 3).

**Table 3:** Mean Reaction time and SD of experimental conditions of Experiment 2b.

	Positive		Negative		Neutral	
	Mean	SD	Mean	SD	Mean	SD
I. Near	451.6	88.5	462.3	99.6	449.8	81.9
II. Middle	564.4	155.8	583.4	140.8	560.0	136.7
III. Far	565.9	134.1	533.1	117.6	540.9	114.9

A two-way ANOVA, 3×3 (region × valence) of mean accuracy data showed no significant interaction, *F*(4, 88) = 0.801, *p* = .53 but main effects of both position, *F*(2, 44) = 22.232, *p*<.001, η^2^_ p_ = .503 and valence, *F*(2, 44) = 6.678, *p*<.01, η^2^_ p_ = .235. Overall, participants were less accurate on positive objects. Bonferroni post-hoc test revealed less accuracy for region II. Detailed analyses of false alarms in region II (38%) confirmed that the amount of false alarms was significantly higher for positive objects as compared to negative and neutral objects, *F*(2,44) = 6.40, *p*<.01, η^2^_ p_ = .225. This suggests a tendency to perceive positive objects as closer than negative or neutral objects (see table 4).

**Table 4:** Mean percentage of correct “yes” responses in region I, percentage of correct “no” responses in region III, and percentage of correct “yes” and “no” responses to region II.

	Positive		Negative		Neutral	
	Mean	SD	Mean	SD	Mean	SD
I. Near	77.9	23.9	77.7	24.7	78.8	24.2
II. Middle	50.4	22.0	52.9	21.6	52.6	21.8
III. Far	83.3	15.8	85.9	14.7	85.9	15.3

A separate test comparing an object’s graspability over RTs did not show any significant main effect of graspability factor, *F*(2,42) = 2.27, *p* = .11, η^2^_ p_ = .097 nor in its interaction with distance, *F*(4,84) = 1.47, *p* = .21, η^2^_ p_ = .065. The same results were observed for the accuracy data: no main effect of graspability, *F*(2,44) = 2.52, *p* = .09, η^2^_ p_ = .010 and no distance × graspability interaction, *F*(4,88) = 1.87, *p* = .12, η^2^_ p_ = .078.

## Discussion

In two experiments we examined how knowledge about the functional and affective properties of objects influenced reaching estimates. In our pilot experiment (Experiment 1), participants sat at a table and were asked to indicate with a verbal response the point at which they thought they could reach several objects that were slowly approached by the experimenter. Manipulations of object type and position were designed to measure the effects of desirability, function familiarity and body posture. Similar to the results of previous studies, participants overestimated their overall reaching capacity [32]. Also consistent with previous studies, our results showed that the orientation of a functional object (e.g. tea jug) affected estimates of reaching distance to that object [25] such that an object in an orientation that made it easy to grasp was perceived as closer than when it was presented in a difficult grasping orientation. Importantly, as pointed out by an anonymous reviewer, and as mentioned above, different motor plans for grasping the various objects could have influenced reaching distance estimation. MacNair and Harris [29], for example, have demonstrated that naming accuracy can be primed by grasping similarities and not by the functional properties of objects. Our results on graspability ratings, however, do not support this assertion. Despite having statistically grouped objects as fine and gross grasping, this variable did not interact with reaching estimate ratios. The result from this pilot experiment therefore supports the idea that perceived reaching distance incorporates knowledge about the details of the actions related to them; the influence of other factors did not emerge as statistically significant. However, the behavioral results obtained in previous studies do suggest that some factors could have been contaminated by the role of the experimenter. As mentioned above, interaction with objects [19] and also property [36] have an important effect on the representation of proximity of objects in the space. In our experiment it was the experimenter who only had contact with the objects and it may be possible that this situation affected participantś internal simulation of reaching. Additionally, previous studies have shown that reaching estimates of objects in movement is affected by the speed of approach [40], which was difficult to control in Experiment 1. Despite these limitations, however, the result contributes to the *action-specific account* [26] by showing that the functional properties of objects influence the perceptual processes of visuo-spatial information.

In Experiment 2, we focused on the examination of the role of the affective properties of objects in reaching estimates. In a long display digital scenario, participants were asked to make similar reaching estimates. Objects appeared on a large horizontal screen controlled by the PC to avoid any influence of the experimenter. Here, our results clearly demonstrated that the affective properties of objects influence perceived reaching. The large amount of false alarms for non-reachable positive objects and the increased RT when such objects were correctly perceived as non-reachable suggests an extension of the peripersonal space mapping, such as that obtained with tools [15]–[17].

It is important to note that participants were instructed not to reach the objects but just to indicate if they thought they could do so. Affective valence biased reaching distance perception at positions near the real reaching limit, when it was difficult to estimate their reaching distance and attention was explicitly focused on the mental simulation of that action. We assume that the differences in proximity estimates found here are a reflection of the role of affective properties of objects and endogenous attention in the action-specific mapping of the peripersonal space [7]. Previous results have demonstrated that “reachability is a metric for perceived distance only when the perceiver intends to reach” [42] p.886. Here the affective properties of objects have influenced reaching estimates even when no reaching action was performed, but in consonance with other studies [30] we instructed participants to imagine they wanted to reach the objects. As no action was required, the observed bias in our reaching estimates suggests that *incentive saliency* [39] effects might also take place during action simulation processes. Experimental evidence found in previous work [25], [19] suggests that grasping properties of objects could have an important effect on reaching estimates. The differences in perceived reaching distance may also be due to several perceptual and motor features of the objects. We analyzed the effects of graspability (fine vs. gross grasping) of objects in our experiments and did not find any significant interactions with distance estimates. It may well be that the fine vs. gross distinction presented here is not sensible enough and that other aspects of the grasping motor plan (e.g. speed) or in the perceptual properties of the objects (e.g. weight) interact with reaching distance estimation. Although our line of research is mainly concerned with the role of emotional properties in spatial cognition and object interaction, these possibilities would be very interesting as a topic of future investigations.

In summary, our results demonstrate that psychological properties of objects such as affective valence and knowledge about their function do indeed influence visual processing involved in action planning even when no explicit action is required. A complete explanation of the variables that determine the peripersonal space mapping should include a more thorough examination of all stages of human-object interaction; the experiments presented in this study addressed only a few. Direct questions that remain to be explored are the role of affective properties at later stages -during action execution, for example - and if the effects observed here in perceived reaching exist only at a functional level or also in the brain activation of the near space system. It is also important to mention a recent finding published by Ambrosini, Scorolli, Borghi, and Costantini [43] in which they report differential effects for actual vs. perceived reaching distance. In their study, verb-object priming effects were significant in the actual reaching space but not in the subjectively perceived reaching space. These results along with previous work [44], suggest that the representation of peripersonal space incorporates implicit connections between our bodies and the actions we execute with them. In our study, 3 participants had a shorter actual reach which resulted in turn a slightly larger amount of false alarms and “No” responses. It may well be that the response dynamics in these subjects interact differently with the affective valence. This opens a very interesting issue that remains to be explored in more detail: namely, that individual differences, and their possible effects in explicit and implicit reaching space perception must be taken into account.

Here we claim that the peripersonal space mapping, indirectly measured from perceived reaching, is affected by the intentions and attention to actions as suggested by the *action-specific account* [26], [7] and also by knowledge about functional and affective properties of the objects involved in such actions. The perceived reaching bias observed with positive objects fits well into the *incentive salience theory* [39]. We propose that the overestimations produced by positive objects reflect the influence of emotional and motivational states in the representation of object-directed actions in the near/far spatial system.
